# Expression of telomerase reverse transcriptase in peripheral T‐cell lymphoma

**DOI:** 10.1002/cam4.4200

**Published:** 2021-09-03

**Authors:** Fumiko Arakawa, Hiroaki Miyoshi, Noriaki Yoshida, Kazutaka Nakashima, Yosaku Watatani, Takuya Furuta, Kyohei Yamada, Mayuko Moritsubo, Mai Takeuchi, Eriko Yanagida, Yasumasa Shimasaki, Kei Kohno, Keisuke Kataoka, Koichi Ohshima

**Affiliations:** ^1^ Department of Pathology School of Medicine Kurume University Kurume Japan; ^2^ Department of Clinical Studies Radiation Effects Research Foundation Hiroshima Laboratory Hiroshima Japan; ^3^ Departments of Hematology and Rheumatology Faculty of Medicine Kindai University Hospital Osaka Japan; ^4^ Division of Hematology Department of Medicine School of Medicine Keio University Tokyo Japan

**Keywords:** copy number assay, immunohistochemistry, mutation analysis, peripheral T‐cell lymphoma, telomerase reverse transcriptase

## Abstract

Telomere length is maintained by the activation of telomerase, which causes continuous cell division and proliferation in many carcinomas. A catalytic reverse transcriptase protein (TERT) encoded by the *TERT* gene plays a critical role in the activation of telomerase. We performed a molecular and pathological analysis of the TERT against three different peripheral T‐cell lymphoma (PTCL) subtypes: PTCL, not otherwise specified (PTCL‐NOS), angioimmunoblastic T‐cell lymphoma (AITL), and adult T‐cell leukemia/lymphoma (ATLL). Immunohistochemical analysis demonstrated TERT expression in 31% of AITL, 11% of PTCL‐NOS, and 5% of ATLL. Among them, AITL frequently showed high TERT expression with statistical significance. TERT promoter mutation analysis and genomic copy number evaluation were performed. TERT promoter mutation was observed in two cases of PTCL‐NOS (2/40) and not in other PTCLs. Genome copy number amplification was detected in 33% of PTCL‐NOS, 33% of AITL, and 50% of ATLL cases. We evaluated the relationship between the analyzed TERT genomic abnormalities and protein expression; however, no apparent relationship was observed. Furthermore, immunostaining showed TERT expression in the PTCL cytoplasm, suggesting the existence of mechanisms other than the maintenance of telomere length. Statistical analysis of the effect of TERT expression on the prognosis in PTCL cases revealed that TERT expression tended to have a poor prognosis in PTCL‐NOS. Since TERT expression was not an independent factor in multivariate analysis, further research will be needed to clarify the poor prognosis of PTCL‐NOS in TERT expression.

## Graphical abstract

In this study, we performed TERT protein expression, mutation analysis of promoter region, and copy number analysis (chr.5) for PTCL (PTCL‐NOS, AITL, ATLL). In AITL, TERT expression was high, but there was no clear relationship between TERT protein expression and genomic abnormality. In the statistical analysis of TERT expression and PTCL prognosis, only PTCL‐NOS expressing TERT had a poor prognosis.

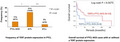

## 1 INTRODUCTION

A telomere is located at the ends of the chromosome, and its length decreases with each cell division.^1^ Telomere shortening blocks further cell division and induces cellular senescence in normal cells.^2^ Telomerase prolongs telomeres by synthesizing terminal telomeric repeats, which maintains cell division ability. There are two important telomerase components: the telomerase RNA that works as an RNA template and the catalytic reverse transcriptase protein (TERT). Cancer cells are characterized by continuous cell division and it is known that the telomere length is stabilized by telomerase in many malignant tumors.^3^ The ectopic expression of TERT causes the immortalization of normal cells in in vitro models.^4^ From this standpoint, TERT expression is considered an important factor for carcinogenesis. TERT expression is commonly silenced in somatic cells; however, it is observed during the embryonic period and in some stem cells, especially in cancer cells.^5^ Somatic mutations (TERT promoter mutations) are often detected at the proximal promoter site of TERT in cancer cells. These mutations cause deregulation of TERT expression, resulting in overexpression. TERT promoter mutations are frequently observed in malignant melanoma, bladder cancer, renal pelvis cancer, thyroid cancer, hepatocellular carcinoma, and malignant glioblastoma. Moreover, it is rarely identified in lung, breast, colon, and gastric cancers.^2^, ^6^, ^7^ A previous study found that the effect of TERT promoter mutation on the transcriptional regulation varies in each cell differentiation stage, suggesting that the difference in TERT promoter mutation frequency is responsible for the difference in cell‐of‐origin of cancer cells.^8^

Non‐Hodgkin lymphoma (NHL) is a malignant tumor that arises from B cells or T/NK cells. These immune cells require clonal proliferation of cells that respond to specific antigens as well as cell division. Therefore, in some mature immune cells, the expression of TERT is maintained so that the cells can continuously proliferate.^9^ In B‐cell lymphoma of NHL, TERT promoter mutations are observed in Mantle cell lymphoma,^10^ but are rare in the other subtypes including diffuse large B‐cell lymphoma which is the most common type of NHL.^11^ In peripheral T‐cell lymphoma, not otherwise specified (PTCL‐NOS), adult T‐cell leukemia/lymphoma (ATLL), and angioimmunoblastic T‐cell lymphoma (AITL) are common subtypes.^12^, ^13^, ^14^ ATLL is linked to the human T‐cell lymphotropic virus type 1 (HTLV‐1) infection, where HTLV‐1 basic leucine zipper (HBZ) is reported to induce the expression of TERT.^15^ However, TERT expression has not been fully investigated in other PTCL disease types.

In this study, we analyzed TERT expression in PTCL‐NOS, ATLL, and AITL and attempted to examine genomic abnormalities related to TERT upregulation in these diseases.

## 2 MATERIALS AND METHODS

### 2.1 Patients

We reviewed 200 PTCL cases composed of 73 PTCL‐NOS, 49 AITL, and 78 ATLL. This study included cases from the International Peripheral T‐cell and Natural Killer/T cell Lymphoma Study^12^ and diagnosed at Kurume University between 2005 and 2019. PTCL‐NOS and ATLL cases were included in our previous studies.^16^, ^17^, ^18^, ^19^, ^20^, ^21^ All cases were reviewed by experienced hematopathologists (O. K., M. H.) according to the World Health Organization classification.^22^ Clinical information was collected by reviewing the patients’ medical charts. Among the analyzed PTCL‐NOS cases in the current study, 27 cases were classified from the presence of genomic alterations in a previous study.^23^ The study was approved by the Research Ethics Committee of Kurume University and was conducted in accordance with the Declaration of Helsinki.

### 2.2 Morphological evaluation

For morphological and immunohistochemical analyses, a tissue microarray (TMA) slide was created, as previously described.^16^, ^17^, ^18^, ^24^ Briefly, the representative tumor areas were identified on the corresponding hematoxylin‐ and eosin‐stained slides. For every case, one sample with approximately 2–3 mm diameter from the paraffin section was obtained using a tissue microarrayer.

Each sample was investigated for its morphologic characteristics according to our previous study.^17^ Neoplastic T cells were detected by assessing morphological findings, including cell size and nuclear atypia. Some evaluated nuclear atypia characteristics include pleomorphic nuclei of varying sizes, large nucleoli, and hyperchromatism with the coarse and irregular distribution.

### 2.3 Immunohistochemical analysis

Immunostaining of TERT was performed using rabbit monoclonal to telomerase reverse transcriptase antibody (Y182) (Abcam). This monoclonal antibody was prepared using a synthetic peptide of the human telomerase reverse transcriptase aa1100‐1200 (C‐terminal) as an immunogen. The conditions for immunostaining were performed as follows. Antigen retrieval was performed by heat mediation at 95°C for 20 min using a microwave. Samples were incubated with primary antibody (1/100) for 30 min. An undiluted HRP‐conjugated goat anti‐rabbit IgG polyclonal was used as the secondary antibody. Tumor cells with more than 30% staining were considered positive in the current study. Immunohistochemical investigation of TBX21 (4B10; Abcam) and GATA3 (D13C9; Cell Signaling Technology) was performed for PTCL‐NOS. Tumor cells with more than 30% staining were considered positive as in our previous study.^24^

### 2.4 PCR and sequencing for *TERT*

Among the samples analyzed using TERT immunostaining, purified DNA from 40 PTCL‐NOS, 21 AITL, and 10 ATLL cases were subjected to TERT promoter mutation analysis. Each of these DNA samples was extracted from frozen sections.

PCR was performed in the region containing the 5’ upstream position −124 and −146 bp from the ATG start codon, which is a hot spot of TERT promoter mutation. Briefly, PCR conditions were as follows: after the initial denaturation at 95°C for 10 min, 40 cycles of 95°C were performed for 30 s, followed by 65°C for 30 s, and 72°C for 30 s, with a final extension at 72°C for 10 min, using AmpliTaq Gold DNA Polymerase Master Mix 360 (Applied Biosystems). The primer sequences were as follows: sense primer, TERT‐P‐FW; 5ꞌ‐cacccgtcctgccccttcaccttcc‐3ꞌ and anti‐sense primer TERT‐P‐RV; 5ꞌ‐ggcttcccacgtgcgcagcagga‐3ꞌ, as used in a previous study.^25^ Amplified PCR products were evaluated in 3% agarose gel and visualized using ethidium bromide staining under ultraviolet light. PCR product sizes were 193 bp.

For the amplified PCR product cleanup, DNA was used with ExoSAP‐IT or ExoSap‐IT Express (Applied Biosystems). Next, Sanger sequencing was conducted to confirm the mutation of the TERT promoter. The direct sequencing reaction was carried out with an ABI Big Dye Terminator v1.1 cycle sequencing kit (Applied Biosystems), cycled at 96°C for 1 min, 25 cycles of 96°C for 10 s, 50°C for 5 s, and 60°C for 4 min. The primer TERT‐P‐FW was used for the sequence reaction. The resulting products were run on an ABI PRISM 310 or SeqStudio Genetic Analyzer (Applied Biosystems).

### 2.5 TaqMan copy number assay for *TERT*

The copy number of hTERT (Hs03078158.cn) was quantified with TaqMan Copy Number Assays (Applied Biosystems) using the same DNA that was subjected to the sequence analysis above. The human RNase P was used as the TaqMan Reference Copy Number Assay (Applied Biosystems). Quantitative real‐time PCR was performed with an ABI 7500 real‐time system (Applied Biosystems) using TaqMan Genotyping Master Mix (Applied Biosystems). The relative copy number of hTERT was calculated with CopyCaller v2.1 software (Applied Biosystems) using the ^ΔΔ^Ct method. A relative copy number >2 was defined as the TERT copy number gain.

### 2.6 Statistical analysis

Frequencies of TERT expression were evaluated using Fisher's exact test or the test with Bonferroni correlation when needed. The overall survival (OS) curves were calculated using the Kaplan–Meier method. The endpoints of OS were defined as the time of relapse and death. A log‐rank test was used to compare survival curves. A Cox proportional hazards model was used in the multivariate analysis. Statistical analyses in this study were carried out with JMP, version 13 (SAS Institute). A *p*‐value of <0.05 indicated statistical significance.

## 3 RESULTS

### 3.1 TERT expression, gene mutation, and copy number variations analysis in PTCL

**FIGURE 1:**
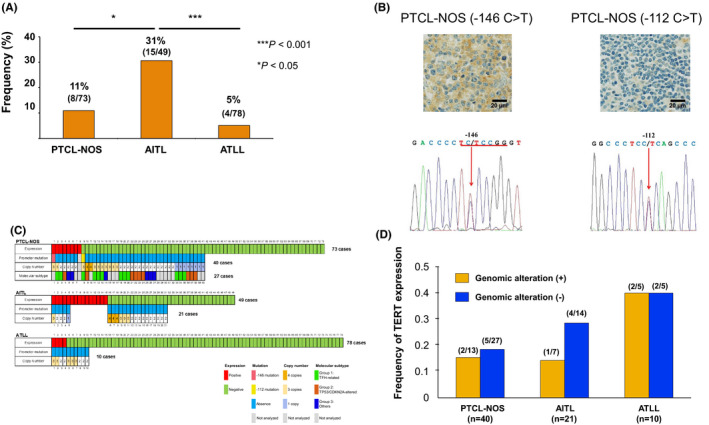
Expression and genomic alterations of TERT in peripheral T‐cell lymphoma (PTCL). (A) Frequency of TERT protein expression in PTCL. The differences were evaluated using Fisher's exact test with Bonferroni correlation. *p < 0.05, ***p < 0.001. (B) Pathological findings and chromatograms of the TERT promoter mutation site in two PTCL‐NOS cases. Red arrow indicates a mutated nucleotide. (C) Association between TERT genomic alterations and the expression. Molecular subtypes are depicted in a part of PTCL‐NOS cases. (D) Frequency of the TERT expression based on genomic alterations in PTCL

We first assessed the TERT expression by immunostaining of each PTCL subtype. Expression of TERT was observed in 8 PTCL‐NOS (11%), 15 AITL (31%), and 4 ATLL (5%) cases (Figure 1A,C). The expression was localized in the cytoplasm in all cases (Figure 1B). Gene mutation analysis of the TERT promoter region was performed to evaluate the expression mechanism of TERT (40 PTCL‐NOS, 21 AITL, and 10 ATLL cases). In this analysis, mutations at the TERT promoter site were found in two PTCL‐NOS cases, but not in other cases. Among the identified mutations, one was −146 bp upstream (−146 C > T) and the other was −112 bp upstream (−112 C > T) from the ATG start site. In addition to somatic mutations, the copy number gain of TERT has also been suggested to be involved in the expression of several tumors.^26^ We, therefore, carried out a copy number assay for TERT in this study. The TERT copy number gain was detected in 13 PTCL‐NOS (33%), 7 AITL (33%), and 5 ATLL (50%) cases (Figure 1C). Two PTCL‐NOS cases with a TERT promoter mutation simultaneously obtained a copy number gain. Therefore, TERT copy number gain was observed in a total of 13 PTCL‐NOS (33%), 7 AITL (33%), and 5 ATLL (50%) cases.

### 3.2 Relationship between the TERT expression and genomic abnormalities

We evaluated the relationship between the TERT expression and genomic abnormalities from PTCL analyses (Figure 1C). In this study, the TERT promoter mutations were present in 2 cases of PTCL‐NOS (Figure 1B). One case had the −146 C > T mutation and was positive for TERT protein. This gene mutation has been shown to enhance TERT promoter activity.^27^, ^28^ Moreover, for −112 C > T, a novel mutation, TERT expression was not observed in the PTCL‐NOS case. Therefore, it is thought that the mutation did not contribute to the TERT expression. We evaluated TERT expression frequency based on genomic abnormalities (Figure 1D). Although the number of cases used for genomic abnormality analysis was small in ATLL, no clear relationship was found between the presence or absence of genomic abnormality and TERT expression (Figure 1D).

### 3.3 TERT expression in subtypes of PTCL‐NOS

Several studies including us have classified PTCL‐NOS, which is a heterogeneous disease, into subtypes based on molecular and pathological findings.^29^ We evaluated TERT expression in the subtypes of PTCL‐NOS. Molecular analysis classified PTCL‐NOS cases into three groups; those with TFH‐related alterations (*TET2*, *RHOA G17V*, and *IDH2*) (Group 1), those with *TP53*/*CDKN2A* alterations (Group 2) and, those lacking any of the above alterations (Group 3).^23^ Among the analyzed PTCL‐NOS cases in the current study, 27 cases were classified from the molecular aspects. Twelve (45%) cases were classified as Group 1, nine (33%) cases were as Group 2, and six (22%) were as Group 3 (Figure 1C). Among the Groups, Group 3 showed a high frequency of TERT expression (33%) compared with the other Groups (Group 1; 17% and Group 2; 11%) (Figure 1C) though the difference was not significant (*p* = 0.676). Additionally, PTCL‐NOS is divided into three subtypes based on the presence of key transcriptional factors for T‐cells; TBX21 and GATA3 and related proteins by immunohistochemistry.^30^ We checked the association between the transcriptional factors and TERT expressions (Table 1). The analysis found that TERT expression was associated with the absence of GATA3 expression (*p* = 0.041) though the expression was not with TBX21 expression (*p* = 0.669).

**TABLE 1:** Statistical association of TERT protein with T‐bet/TBX21 and GATA3 in PTCL‐NOS

	TERT + (8)	TERT – (64)	*p*‐value
T‐bet/TBX21 + (15)	2 (25.0%)	13 (20.3%)	0.669
T‐bet/TBX21 ‐ (57)	6 (75.0%)	51 (79.7%)	
GATA3 + (58)	4 (50.0%)	54 (84.4%)	0.041
GATA3 – (14)	4 (50.0%)	10 (15.6%)	

### 3.4 Clinical significance of TERT expression

**FIGURE 2:**
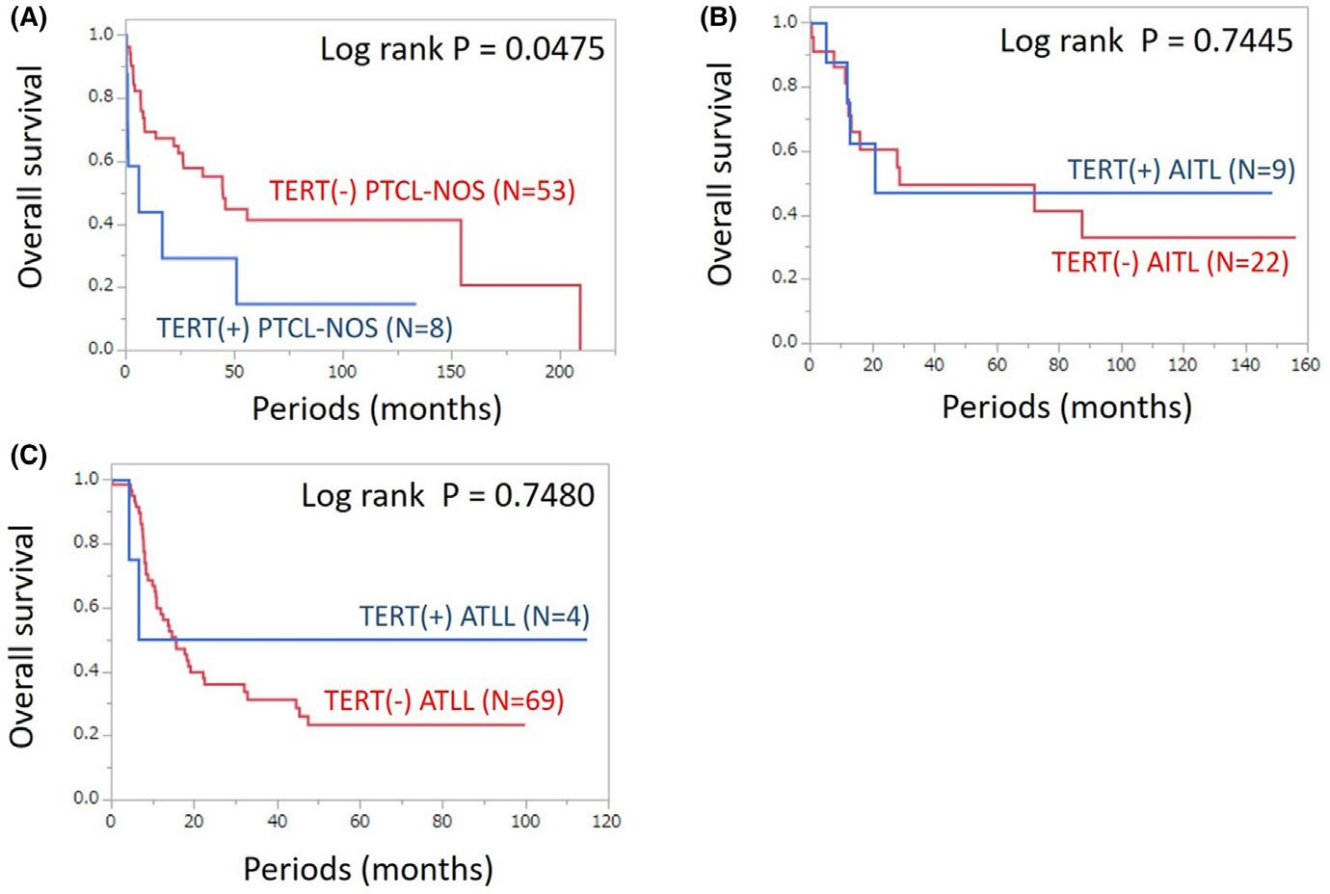
Overall survival of the cases with or without a TERT expression. A log‐rank test was used to compare survival curves. (A) PTCL‐NOS; (B) AITL; (C) ATLL

TERT promoter mutations and their expression have been reported as prognostic factors in several tumors, including glioblastoma and malignant melanoma.^31^, ^32^, ^33^, ^34^ Since the TERT expression in PTCL is thought to be controlled by various mechanisms other than the promoter site mutations, we investigated the clinical impact of TERT protein expression in our PTCL cases (Figure 2). In PTCL‐NOS, TERT expression was associated with a poor prognosis and a statistical significance (Median overall survival, TERT‐positive 6.2 Months, 95% confidence interval [CI] 0.5–51.1, TERT‐negative 45 months, 95% CI 22.3–209.1; *p* = 0.0475, Log‐rank test). This trend was not observed for AITL and ATLL. Since the international prognostic index (IPI), presence of B‐symptoms, and the expression of cytotoxic molecules are currently recognized as prognostic factors in PTCL‐NOS,^35^, ^36^, ^37^, ^38^ we simultaneously integrated these known prognostic markers and TERT expression in a multivariate analysis to show the risk of each factor (Table 2). The analysis found that IPI was an independent poor prognostic factor; however, other factors, including TERT expression, were not independent prognostic factors.

**TABLE 2:** Multivariate analysis for overall survival in patients with PTCL‐NOS

	HR	95%CI	*p*‐value
IPI >1	3.53	1.13–10.96	0.0294
Expression of cytotoxic molecules	0.74	0.23–2.36	0.614
Presence of B‐symptom	1.09	0.34–3.49	0.884
Presence of TERT expression	1.7	0.29–9.97	0.555

Abbreviations: CI, confidence interval; HR, hazard ratio; IPI, international prognostic index; PTCL‐NOS, peripheral T‐cell lymphoma, not otherwise specified.

## 4 DISCUSSION

In this study, we analyzed the TERT expression in PTCL and its genomic abnormalities. The expression of TERT protein was most frequently observed in AITL among the PTCL cases examined. We also found that TERT promoter mutations were rare in PTCL. Regarding TERT promoter mutations, it is known that −146 C > T mutation enhances TERT promoter activity by taking the TTCCGG sequence.^27^, ^28^ Indeed, the PTCL‐NOS case with the −146 C > T mutation was positive for TERT protein. In the other PTCL‐NOS case, −112 C > T mutation did not result in the TTCCGG sequence. Therefore, it is thought that the mutation did not contribute to the TERT expression.

Cases with a copy number gain of TERT were not consistently positive for TERT protein (Figure 1C). This finding is in line with previous results of a part of cancers.^39^ A possible explanation for the lack of association is that the probes for *TERT* copy number analysis in the studies including the current study do not cover the entire *TERT* gene. Additionally, TERT expression has been known to be regulated by several mechanisms including transcription, splicing, epigenetics,^40^ and this tight regulation mechanism may be also related to the lack of association between copy number gain and the expression in TERT.

In normal T cells, it has been reported that the telomere length is shorter, and the frequency of cell division is lower in memory T‐cells than in naïve T cells.^41^ AITL, which showed a high frequency of the TERT expression in the current study, is considered a tumor derived from follicular helper T‐cells.^22^ One of the pathological characteristics of AITL is the presence of the Epstein‐Barr virus (EBV) infection in non‐tumor cells. EBV‐reactive peripheral CD8‐positive T‐cells have been reported to express telomerase,^42^ and a similar mechanism may exist in AITL. A previous study showed that T‐cells from both young and old donors can re‐express telomerase after anti‐CD3/CD28 stimulation, suggesting the importance of T‐cell receptor (TCR) signaling in the maintenance of telomeres in T‐cells.^43^ In addition, TERT phosphorylated by the NF‐κB activation appears to translocate from the cytoplasm into the nucleus of normal T‐cells.^44^ All positive cases in this analysis were also positive in the cytoplasm, and no translocation into the nucleus was observed. Large‐scale genomic aberration analysis has revealed that the activation of TCR signaling plays a critical role in the pathophysiology of PTCL.^23^, ^45^, ^46^, ^47^, ^48^ RHOA G17V mutation was found in approximately 70% of AITL cases, and the mutation causes the activation of TCR signaling.^49^, ^50^ Therefore, it is speculated that the analyzed AITL cases also have the activation of the TCR signaling pathway, but TERT was found in the cytoplasm. These findings suggest that TERT plays different roles in PTCL other than the maintenance of telomere length. In addition to telomere DNA synthesis activity, TERT can play as an RNA‐dependent RNA polymerase.^51^ It is also found that the activity of RNA‐dependent RNA polymerase was associated with the maintenance of cancer stem cells^52^ and that the RNA polymerase can control several gene expressions.^53^ Furthermore, it is reported that TERT localized in mitochondria shuttled from the nucleus under oxidative stress,^54^ and it protects mitochondrial function. This prevents DNA damage by the reduction of reactive oxygen stress from mitochondria and it is considered that this mechanism is involved in resistance to chemotherapies in cancer cells. As we found that TERT expression in PTCL was mainly found in the cytoplasm, we speculate that those mechanisms contribute to the pathogenesis of PTCL that is recognized as a dismal disease.^55^

In this analysis, the TERT expression was observed in 11% of PTCL‐NOS. PTCL‐NOS is a heterogeneous disease that does not belong to other PTCLs. Some PTCL‐NOS cases have been shown to be AITL‐like diseases based on cell surface markers and genomic mutations.^23^ PTCL‐NOS expressing the AITL surface antigen markers PD‐1, CD10, CXCL13, and BCL6 were excluded in this analysis and further analysis of PTCL‐NOS cases and other types of PTCL may precisely elucidate the significance of the TERT expression in PTCL.

In ATLL, HBZ of HTLV‐1 and JunD has been reported to enhance TERT transcriptional activity.^15^ Moreover, Tax, which is another viral protein of HTLV‐1, suppresses TERT along with T‐cell acute lymphoblastic leukemia 1 (TAL1), which causes genomic instability in HTLV‐1‐infected cells. Although Tax is silenced by methylation in many ATLLs, HBZ expression is observed in such ATLLs.^56^ We previously analyzed the existence of HBZ and Tax in ATLL by RNA in situ.^21^ Although some of the cases analyzed in this study (42 ATLL cases) were also included in the previous study, we could not assess the relationship between TERT and HBZ expression due to the limited number of TERT positive cases (two cases) in ATLL (data not shown). The fact that the expression of HBZ was observed in all 42 cases supports that the expression of TERT in ATLL may also be caused and maintained by other mechanisms.

The molecular classification found that there was no clear association between TERT expression and the molecular subtype in PTCL‐NOS though the analyzed cases were limited. Regarding the cell‐of‐origin, we analyzed the expressions of TBX21 and GATA3. We found that TERT expression was associated with the absence of GATA3 expression though the expression was not with TBX21 expression, suggesting that the cases with TERT expression are not classified as PTCL‐GATA3 group at least.

Although this study showed the significance of TERT in PTCLs, there are some limitations in this study. First, this study includes only three subtypes of PTCL. Other subtypes including ALCL including nodular PTCL with Tfh phenotypes should be investigated in the future to clarify the role of TERT in PTCLs. Second, the validity of the cut‐off value at 30% for IHC of TERT protein is still unknown. Third, a validation cohort using more samples will be required to show the significance.

In this study, the TERT expression was associated with a poor prognosis in PTCL‐NOS but was confounded with other factors in the risk analysis. Recently, small‐molecule inhibitors for TERT or telomerase RNA have been developed and showed a response in several cancers.^57^ Such inhibitors may also be effective in TERT‐positive PTCL and may improve the prognosis.

## CONFLICT OF INTEREST

The authors declare no potential conflicts of interest.

## AUTHOR CONTRIBUTION

Conception and design: FA and NY. Acquisition of data (acquired and managed patients, etc.): HM, MM, MT, EY, KK, YS, and KO. Analysis and interpretation of data (e.g., immunohistochemistry, sequence analysis, statistical analysis, computational analysis): FA, HM, KN, TF, and KY. Analysis of molecular subclass for PTCL‐NOS: YW and KK. Writing and review of the manuscript: FA, NY, and HM. Study supervision: HM and KO.

## ETHICAL APPROVAL STATEMENT

The study was approved by the Research Ethics Committee of Kurume University and was conducted in accordance with the Declaration of Helsinki.
